# The *RyR2-P2328S* mutation downregulates Na_v_1.5 producing arrhythmic substrate in murine ventricles

**DOI:** 10.1007/s00424-015-1750-0

**Published:** 2015-11-06

**Authors:** Feifei Ning, Ling Luo, Shiraz Ahmad, Haseeb Valli, Kamalan Jeevaratnam, Tingzhong Wang, Laila Guzadhur, Dandan Yang, James A. Fraser, Christopher L.-H. Huang, Aiqun Ma, Samantha C. Salvage

**Affiliations:** Department of Cardiovascular Medicine, First Affiliated Hospital of Xi’an Jiaotong University, No. 277 West Yanta Road, Xi’an, Shaanxi 710061 People’s Republic of China; Physiological Laboratory, University of Cambridge, Cambridge, CB2 3EG UK; Faculty of Health and Medical Science, Duke of Kent Building, University of Surrey, Guildford, GU2 7TE UK; Perdana University-Royal College of Surgeons Ireland, 43400 Serdang, Selangor Darul Ehsan, Malaysia; Key Laboratory of Molecular Cardiology, Shaanxi Province, People’s Republic of China; Key Laboratory of Environment and Genes Related to Diseases (Xi’an Jiaotong University), Ministry of Education, Xi’an, People’s Republic of China; Department of Biochemistry, University of Cambridge, Cambridge, CB2 1QW UK; Niche Science and Technology, Falstaff House, Bardolph Road, Richmond, UK

**Keywords:** RyR2, CPVT, Na_v_1.5, Arrhythmogenicity, Conduction velocity, Ca^2+^ homeostasis

## Abstract

Catecholaminergic polymorphic ventricular tachycardia (CPVT) predisposes to ventricular arrhythmia due to altered Ca^2+^ homeostasis and can arise from ryanodine receptor (*RyR2*) mutations including *RyR2-P2328S*. Previous reports established that homozygotic murine *RyR2*-*P2328S* (*RyR2*^S/S^) hearts show an atrial arrhythmic phenotype associated with reduced action potential (AP) conduction velocity and sodium channel (Na_v_1.5) expression. We now relate *ventricular* arrhythmogenicity and slowed AP conduction in *RyR2*^S/S^ hearts to connexin-43 (Cx43) and Na_v_1.5 expression and Na^+^ current (*I*_Na_). Stimulation protocols applying extrasystolic S2 stimulation following 8 Hz S1 pacing at progressively decremented S1S2 intervals confirmed an arrhythmic tendency despite unchanged ventricular effective refractory periods (VERPs) in Langendorff-perfused *RyR2*^S/S^ hearts. Dynamic pacing imposing S1 stimuli then demonstrated that progressive reductions of basic cycle lengths (BCLs) produced greater reductions in conduction velocity at equivalent BCLs and diastolic intervals in *RyR2*^S/S^ than WT, but comparable changes in AP durations (APD_90_) and their alternans. Western blot analyses demonstrated that Cx43 protein expression in whole ventricles was similar, but Na_v_1.5 expression in both whole tissue and membrane fractions were significantly reduced in *RyR2*^S/S^ compared to wild-type (WT). Loose patch-clamp studies similarly demonstrated reduced *I*_Na_ in *RyR2*^S/S^ ventricles. We thus attribute arrhythmogenesis in *RyR2*^S/S^ ventricles resulting from arrhythmic substrate produced by reduced conduction velocity to downregulated Na_v_1.5 reducing *I*_Na_, despite normal determinants of repolarization and passive conduction. The measured changes were quantitatively compatible with earlier predictions of linear relationships between conduction velocity and the peak *I*_Na_ of the AP but nonlinear relationships between peak *I*_Na_ and maximum Na^+^ permeability.

## Introduction

Catecholaminergic polymorphic ventricular tachycardia (CPVT) is an inherited arrhythmic syndrome characterized by episodic syncope and/or sudden cardiac arrest, typically triggered by adrenergic stimulation as occurs during strenuous exercise or emotional stress [1, 34, 46]. CPVT has been associated with mutations in various Ca^2+^ handling proteins, which lead to abnormalities in Ca^2+^ homeostasis; most notably, these mutations are found in the cardiac ryanodine receptor-Ca^2+^ release channel (RyR2) [25, 35] and the sarcoplasmic reticulum (SR) Ca^2+^-binding protein calsequestrin 2 (CASQ2) [13, 19]. Ca^2+^-dependent calmodulin missense mutations also occur in a small number of cases [18]. Some *RyR2* mutations also predispose to atrial arrhythmias [4, 34, 39]. For example, the *RyR2-P2328S* mutation is associated with high incidences of both CPVT and atrial tachycardia (AT) [25, 37, 40]. This *RyR2* variant has been associated with a normal luminal SR Ca^2+^ release sensitivity but an increased sensitivity to cytosolic Ca^2+^ [31], giving rise to lower cytosolic Ca^2+^ thresholds leading to Ca^2+^ release. If reached during increased heart rates, these could be sufficient to elicit a ‘leak’ of SR Ca^2+^ consequently triggering arrhythmia.

The atrial and ventricular phenotypes are replicated by the *RyR2-P2328S* (*RyR2*^S/S^) murine model which shows potential arrhythmic triggers in the form of delayed after-depolarizations [15, 22]. However, there remains a requirement for an electrophysiological tissue substrate in order to perpetuate and sustain arrhythmia, which has previously been typified by a reduced conduction velocity *(θ*) in systems showing reduced Na_v_1.5 expression [33], connexin 40 and/or 43 (atria) or connexin 43 (ventricle) expression [16, 23], or structural abnormalities, including fibrosis [44]. Interestingly, several reports have indicated the potential for Na_v_1.5 expression [6, 11, 42] and function [2, 41] to be modulated, both directly and indirectly, by alterations in cytosolic Ca^2+^. Rat cardiomyocytes showed reductions in Na^+^ channel activity following imposed increases of intracellular [Ca^2+^]. Additionally, the Ca^2+^ channel blocker verapamil and the Ca^2+^ ionophore calcimycin increased and decreased Na_v_1.5 mRNA and Na_v_1.5 protein expression respectively [11, 42]. In agreement with these findings, elevations and reductions of cytosolic [Ca^2+^], by chronic treatment with high extracellular [Ca^2+^] and [K^+^] or BAPTA-AM, decreased and increased Na^+^ current densities, respectively [6]. More recently, Casini et al. [5] demonstrated that acute increases in pipette [Ca^2+^] were capable of reducing both Na^+^ current density and (d*V*/d*t*)_max_.

Biochemical evidence accounting for the potential mechanisms of functional modulation of Na_v_1.5 by cytosolic [Ca^2+^] identifies both direct and indirect Ca^2+^ binding sites on Na_v_1.5. *Direct* Ca^2+^ binding to Na_v_1.5 is mediated at an EF hand motif resident at the carboxy-terminal region of Na_v_1.5 [47]. This binding results in a depolarizing shift of the voltage dependence of Na^+^ channel inactivation with a potential increase in Na^+^ channel activity [47]. *Indirect* mechanisms of Ca^2+^ binding have been attributed to both the presence of an additional binding site, the ‘IQ’ domain, within the C-terminal region of Na_v_1.5 for Ca^2+^/Calmodulin (Ca^2+^/CaM) and multiple phosphorylatable sites (including serines 516 and 571 and threonine 594) within the IDI-II linker region of Na_v_1.5 targeted by Ca^2+^/CaM Kinase II (CaMKII). These two mechanisms occur only subsequent to Ca^2+^ binding to the EF hand motifs of Ca^2+^/Calmodulin (Ca^2+^/CaM) or Ca^2+^/CaM Kinase II (CaMKII) and thus constitutes an indirect interaction of Ca^2+^ with Na_v_1.5. Ca^2+^/CaM binding at the IQ domain and CaMKII-dependent phosphorylation shifts Na^+^ current availability to a more depolarized membrane potential [2] and enhances slow inactivation of the Na^+^ current [41].

Recent reports have indeed implicated reduced Na_v_1.5 expression and Na^+^ channel function in the increased arrhythmogenicity in *RyR2*^S/S^ atria [21, 22, 36]. They also demonstrated a reduced conduction velocity [22], resulting from a reduced Na^+^ current attributable either to a reduced Na_v_1.5 expression or the direct inhibitory effect on Na^+^ channel function of altered Ca^2+^ homeostasis outlined previously. Slowed conduction resulting from reduced Na_v_1.5 expression would potentially produce arrhythmogenic substrate, which would compound the arrhythmic effect of Ca^2+^-mediated triggered activity in the *RyR2*^S/S^ [21, 22, 36].

The present study investigates for possible roles of Cx43 expression as well as Na_v_1.5 expression and function in *RyR2*^S/S^*ventricular*, as opposed to atrial, arrhythmogenicity. First, the arrhythmogenic properties of the *RyR2*^S/S^ ventricle compared to the WT was confirmed in accordance with earlier reports [15] and correlated with measurements of action potential duration (APD), conduction velocity (*θ*) and their alternans, as well as ventricular effective refractory period (VERP). The stimulation protocols either interposed extrasystolic, S2, stimuli at progressively decremented S1S2 intervals within 8 Hz S1 pulse trains or applied steady stimulus frequencies at progressively decremented basic cycle lengths (BCLs). Second, to assess the underlying mechanism for the slowed conduction and arrhythmic phenotype, we assessed the ventricular expression of Cx43 and Na_v_1.5, the latter assessed in both the whole ventricle and the membrane fraction compared between WT and *RyR2*^S/S^ hearts. Third, the corresponding functional evaluation of Na_v_1.5 was investigated through peak *I*_Na_ current recordings of WT and *RyR2*^S/S^ ventricular tissue. These comparisons successfully correlated Na_v_1.5 expression and function, particularly within the functional Na_v_1.5 containing membrane fraction, with the incidence of ventricular arrhythmia, and the resulting conduction changes in *RyR2*^S/S^ ventricles.

## Materials and methods

### Experimental animals

Homozygous *RyR2*^S/S^ and WT mice (aged 4 to 6 months) with an inbred 129/Sv genetic background (supplied initially by Harlan, UK) were generated as described previously [15]. Mice were kept in plastic cages at room temperature in an animal facility, given free access to sterile rodent chow and water and subjected to 12 h light/dark cycles. Mice were killed by cervical dislocation for experimentation. All procedures conformed to the UK Animals (Scientific Procedures) Act 1986, approved by a university ethics review board. Hearts were rapidly excised and submerged in ice-cold Krebs-Henseleit (KH) buffer solution (containing, in mM, NaCl 119, NaHCO_3_ 25, KCl 4, KH_2_PO_4_ 1.2, MgCl_2_ 1, CaCl_2_ 1.8, glucose 10 and Na-pyruvate 2, pH 7.4, 95 % O_2_/ 5 % CO_2;_ British Oxygen Company, Manchester, UK) for whole heart electrophysiological studies and Western blot analyses. All chemicals were purchased from Sigma-Aldrich (Poole, Dorset, UK), unless otherwise stated. Six WT and seven *RyR2*^S/S^ mice were used in whole heart electrophysiological investigations. Four WT and four RyR2^S/S^ hearts were used for Western blot studies of Cx43 expression. Seven WT and six *RyR2*^S/S^ mice were used for Western blot studies of Na_v_1.5 expression in the whole tissue and membrane fraction. Four WT and three *RyR2*^S/S^ mice were used to give *n* = 6 and 12 patches, respectively, for loose patch-clamp studies of Na^+^ currents. Male and female mice were used in statistically equal numbers in each group.

### Electrophysiological studies in whole heart

Excised hearts were cannulated and retrograde perfused on a Langendorff system as previously described [28, 29, 38, 48]. Prior to electrophysiological testing, hearts were perfused with KH solution for at least 5 min to achieve a steady state. Monophasic action potentials (MAPs) were recorded by microMAP electrodes (HugoSachs, Harvard Apparatus, UK) placed upon the epicardial surface. Recordings were amplified (Neurolog NL100 preamplifier; NL104 amplifier, Digitimer, Welwyn Garden City, UK), band-pass filtered (NL125/126 filter; 0.5 Hz to 1.0 kHz) and sampled at 5 kHz (micro1401 interface) for display using Spike2 software (Cambridge Electronic Design, Cambridge, UK).

**Fig. 1:**
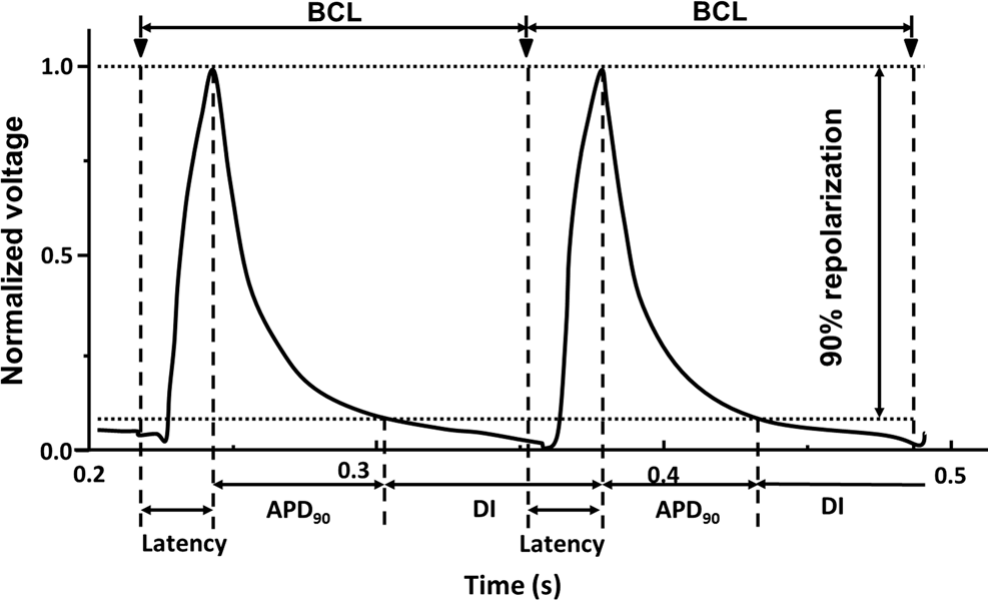
Two typical successive monophasic action potential (MAP) recordings at the LV epicardium of a WT heart obtained during dynamic pacing to highlight the derivation of the various parameters used for analysis; BCL, latency, APD_90_ and DI. BCL was the time interval between the adjacent stimuli, thus the pacing rate. Latency was measured as the time elapsed from the stimuli to the peak of MAP. APD_90_ is the time course over which 90 % repolarization of the MAPs obtained. DI was measured as BCL-APD_90_, thus comprising the final 10 % of MAP repolarization and up to the start of the next stimuli

Hearts were paced at twice their excitation threshold voltage using a bipolar platinum-coated stimulating electrode placed on the ventricular septum connected to a DS2A-Mk.II stimulator (Digitimer). After pacing at 8 Hz for at least 5 min to attain and confirm a steady state, two types of pacing protocols were applied. A S1S2 protocol first stimulated hearts at frequencies of 10 Hz for 20 s; this was followed by cycles of drive trains of eight S1 beats delivered at 8 Hz followed by an S2 extra-stimulus, at S1-S2 coupling interval successively reduced by 1 ms with each subsequent cycle until either 2:1 block or sustained arrhythmia occurred. A dynamic pacing protocol [24, 28, 29] first assessed action potential (AP) properties at a BCLs of 134 ms duration for 100 stimulations. The BCL was then decremented by 5 ms, and the pacing sequence repeated until the hearts showed either entry into 2:1 block or sustained arrhythmia. Both stimulation protocols yielded incidences of arrhythmia. The S1S2 protocol additionally provided VERPs. The dynamic pacing protocol yielded APDs, and an indication of conduction velocity *θ*’ (1/latency, which was measured as the time from the stimuli to the peak of the MAP) as a function of BCL, measured as the recovery time from AP peak to 90 % full repolarization, APD_90_ (Fig. 1). The corresponding diastolic intervals (DIs) were calculated from the BCL and APD_90_ values using the relationship:

$$ \mathrm{D}\mathrm{I}=\mathrm{B}\mathrm{C}\mathrm{L}-{\mathrm{APD}}_{90} $$

### Protein extraction and Western blot analysis

The method of protein extraction was optimized for the protein of interest. For connexin proteins, which are primarily found in the surface membrane in hexameric clusters, a well-established high content sodium dodecyl sulphate (SDS) buffer [7, 12, 43] was used in order to fully solubilize the membrane and maximize release of the connexin proteins from the gap junction channels in the plasma membrane. For Na_v_1.5 channels, we chose to use a milder buffer, followed by a centrifugation step and solubilization in order to separate out the membrane and non-membrane fractions based on the different distribution and abundance of Nav1.5 channels and their contribution to conduction.

Ventricular tissues of the excised hearts were snap-frozen and crushed into powder by a clamp pre-cooled with liquid N_2_. The powdered tissue was homogenized in either SB20 (20 % SDS, 2 mM EDTA, 150 mM Tris) [7, 12, 43] and diluted to an appropriate gel loading concentration in sample loading buffer (90 % SB20, 5 % 2-mercaptoethanol, 5 % *w*/*v* bromophenol blue) for connexin 43 analysis, or the re-suspension buffer (containing, in mM, Tris–HCl 50, NaCl 10, Sucrose 320, EDTA 5, EGTA 2.5 and Protease inhibitors (1 tablet/20 ml; Roche, West Sussex, UK), pH 7.4) and lysis buffer (containing, in mM, Tris–HCl 20, EDTA 2, NaCl 137 and Triton X-100 1 %, glycerol 10 %, pH 7.4) and then centrifuged for 15 min at 13,000*g* and 4 °C for Na_v_1.5 analysis. The supernatant was divided into two parts. One was stored at −80 °C as the whole tissue fraction and the rest was centrifuged at 100,000*g* at 4 °C for an hour to extract the membrane proteins. The pellet was re-suspended in radioimmunoprecipitation assay (RIPA) lysis buffer and then vortexed and placed on ice for 30 min to harvest the membrane protein.

For Western blot analysis, the protein extracts from whole tissue and the membrane fraction were separated on premade 4–12 % Bis-Tris Gels (Invitrogen, Paisley, UK) and then transferred onto PVDF membranes (Immobilon-P, Millipore, Hertfordshire, UK). Blots were blocked in 5 % skimmed milk in TBST (Tris-buffered saline; Invitrogen, Paisley, UK and Tween 20) and then probed with either anti-Na_v_1.5 (rabbit anti-mouse IgG, 1:1000, Alomone, Jeruselam, Israel) or anti-Cx43 (rabbit anti-mouse, 1:10,000, C6219 Sigma-Aldrich) and anti-GAPDH (Abcam, Cambridge, UK) or anti-α-tubulin (Cell Signaling Technology, Danvers, MA, USA) antibodies overnight at 4 °C. Horseradish peroxidase (HRP)-conjugated secondary antibody (Abcam, 1:10000 to 1:50000) were detected using an enhanced chemiluminescent system (GE Healthcare, Little Chalfont, Bucks, UK). Specific protein bands were quantified using Image J (National Institutes of Health, Wash., USA).

### Loose patch clamp recordings of *I*_Na_ in ventricular tissue

Loose patch clamp experiments previously described for atrial tissue [21, 36] were performed in a right ventricular tissue preparation. This technique allows for measurement of Na^+^ currents in whole, perfused ventricular tissue preparations where intracellular Ca^2+^ homeostasis is not disrupted by cell isolation. Furthermore, the tissue preparation allows a more reliable comparison with APD_90_ and CV measurements of this study. Activation properties were assessed in order to determine peak *I*_Na_. The activation protocol utilized a series of 75 ms duration depolarizing pulses, incremented by 10 mv steps ranging from 20 to 120 mV excursions applied 5 ms after the beginning of the sampling period using a P/4 pulse protocol [3].

### Statistical analysis

Statistical analysis for differences between experimental groups was performed using Graphpad Prism software (La Jolla, CA 92037 USA), applying unpaired Student’s *t* tests. A value of *P* < 0.05 was considered statistically significant. Data are presented as means ± SEM.

## Results

### Comparison of ventricular arrhythmogenicity in S1S2 protocols and dynamic protocols

We initially confirmed the arrhythmogenic phenotype of the *RyR2*^S/S^ murine heart as previously reported [15]. An S1S2 stimulation protocol was used to determine the incidence, frequency and duration of ventricular arrhythmia in isolated Langendorff-perfused hearts.

**Fig. 2:**
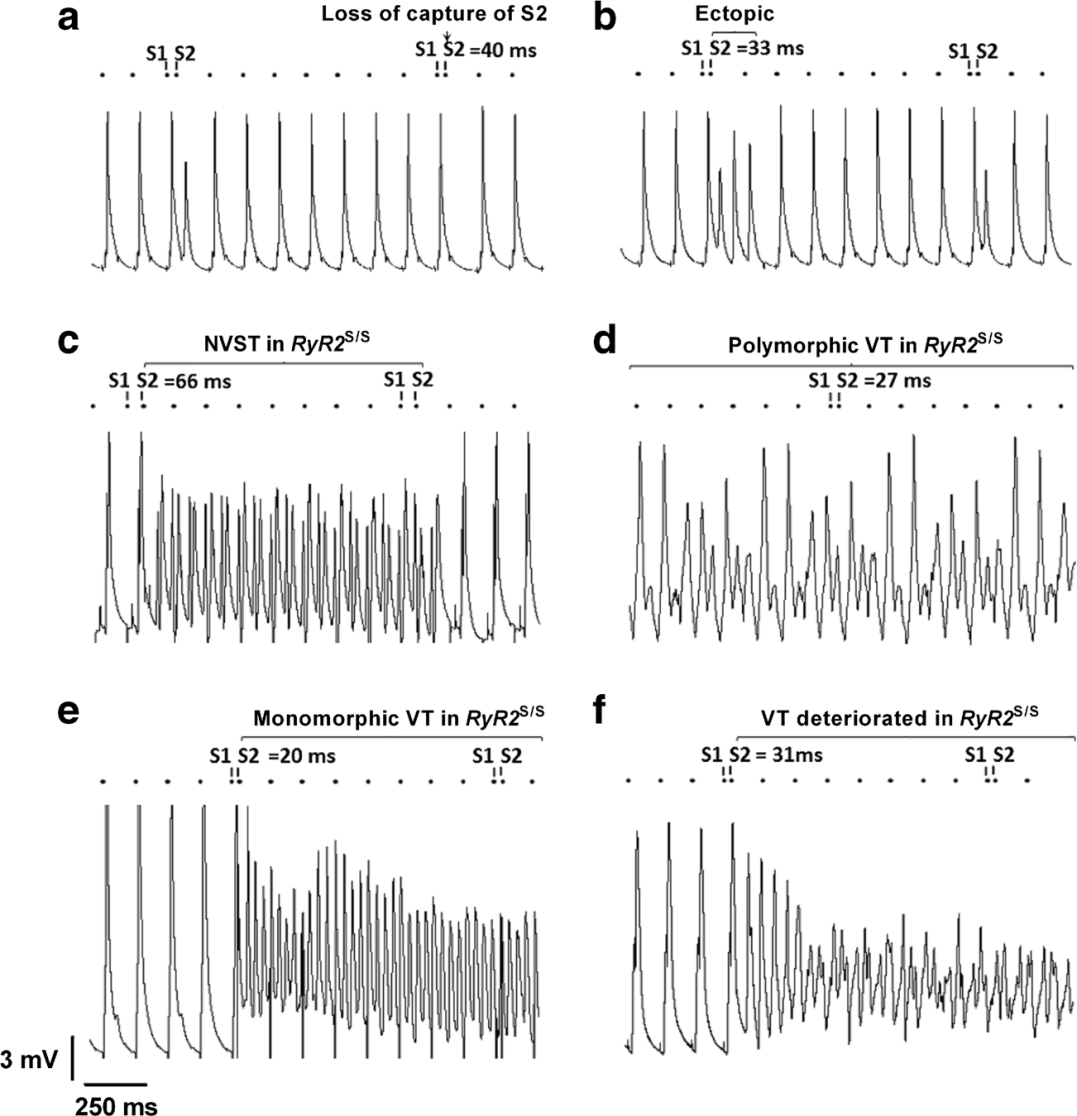
MAP traces obtained from the epicardium of the left ventricle in WT and *RyR2* ^S/S^ hearts during S1S2 pacing highlighting typical traces of either normal activity or arrhythmogenesis. All WT hearts entered the refractory period without displaying any episodes of arrhythmia, as defined by two or more non-stimulated APs (**a**); however, one heart displayed the occurrence of a singular ectopic (one non-stimulated AP) (**b**). Multiple arrhythmic events were observed in *RyR2* ^S/S^ hearts including short non-sustained ventricular tachycardia (NSVT) (**c**), polymorphic tachycardia following a previously imposed S2 extrastimulus (**d**), monomorphic ventricular tachycardia (VT) (**e**) and episodes of VT which deteriorated into ventricular fibrillation (VF) (F). The small black circles indicate the timing of stimuli

The occurrence of ventricular tachycardia (VT) was defined as an occurrence of two or more sequential spontaneous APs, as in previous work [36] in the course of programmed electrical stimulation. Figure 2 illustrates representative left ventricular epicardial traces from WT (A, B) and *RyR2*^S/S^ (C-F) hearts, displaying regular activity (A), ectopic (B) and VT (C-E) and ventricular fibrillation (VF) (F) episodes during the S1S2 protocol. Six WT and seven *RyR2*^S/S^ hearts were subject to the S1S2 protocol described in methods. None of the WT hearts showed VT, although one heart showed three separate ectopic events (at S1S2 intervals of 33, 31 and 30 ms). In contrast, the *RyR*^S/S^ hearts showed 30 episodes of arrhythmia, taking the form of either polymorphic or monomorphic VT in three of the hearts. Of these hearts, the first showed 7 episodes of VT, lasting approximately 2.8 s, with an additional VT episode that degenerated into VF lasting approximately 22.5 s. The second heart showed one episode of VT lasting approximately 11.2 s. The third heart showed 21 episodes of VT accounting for a total time of approximately 14 s.

Ventricular effective refractory periods (VERPs) were defined as the time period during which the myocardium is incapable of re-excitation in response to the twice-threshold stimulus employed in the S1S2 protocols [10, 32]. It was thus the S1S2 interval at which loss of S2 capture first occurred in an absence of VT. WT and *RyR2*^S/S^ hearts typically became refractory at similar S1S2 pacing intervals (VERP: WT: 38.2 ± 1.55 ms (*n* = 6); *RyR2*^S/S^: 37.5 ± 5.04 ms (*n* = 5); *P* = 0.9057). VERP could not be ascertained from all the mice studied due to arrhythmogenesis: sustained arrhythmias occurred in two of the seven *RyR2*^S/S^ hearts during the S1S2 protocol.

The differing arrhythmic properties of WT and *RyR2*^S/S^ were further confirmed in the dynamic pacing protocol, which subjected hearts to systematically decreasing BCLs. Two of the six WT hearts showed VT at BCLs of 39 and 44 ms. However, these correspond to BCLs which are substantially lower, thus a much higher heart rate than those experienced during normal physiological maximal exercise [8]. *RyR2*^S/S^ hearts not only demonstrated higher incidences of VT and VF but they occurred also at higher BCLs than WT (54, 64, 54 and 74 ms in four *RyR2*^S/S^ hearts respectively; these necessitated termination of the protocol), suggesting a reduced capacity to tolerate increased heart rates as may be observed during emotional or physical stress such as exercise [8].

### Action potential properties and conduction at varying pacing rates

**Fig. 3:**
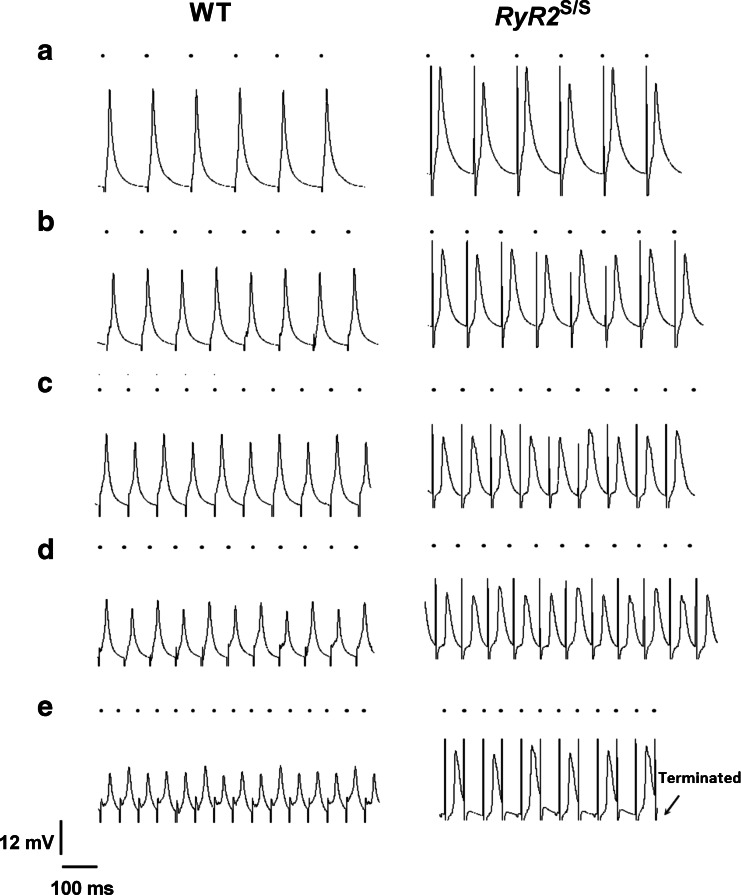
Typical MAP recordings obtained from the left ventricular epicardium of WT and *RyR2* ^S/S^ during dynamic pacing. Traces from WT (*left*) and *RyR2* ^S/S^ at progressively decreasing BCLs: 124 (**a**), 99 (**b**), 84 (**c**), 74 (**d**) and 54 ms (**e**). If a heart entered 2:1 block, the protocol was terminated (E). Traces are displayed along a common horizontal timescale. The vertical scale was normalized to a standard AP deflection at a BCL of 134 ms. *Small black circles* above each trace indicate the timing of stimuli

AP propagation and recovery properties at different BCLs were then investigated using the dynamic pacing protocol. Figure 3 illustrates typical APs thus obtained from the LV epicardium of WT (left column) and *RyR2*^S/S^ hearts (right column). In both cases, AP amplitude decreased with increasing pacing rate in every heart, independent of genotype (Fig. 3a–e). At lower pacing rates, *RyR2*^S/S^ hearts showed a higher incidence of alternans (Fig. 3a, right column) compared with WT hearts (Fig. 3c, left column). Around half of both the WT and the *RyR2*^S/S^ hearts had shown either a loss of capture or arrhythmogenesis when the BCL reached 54 ms (Fig. 3e).

**Fig. 4:**
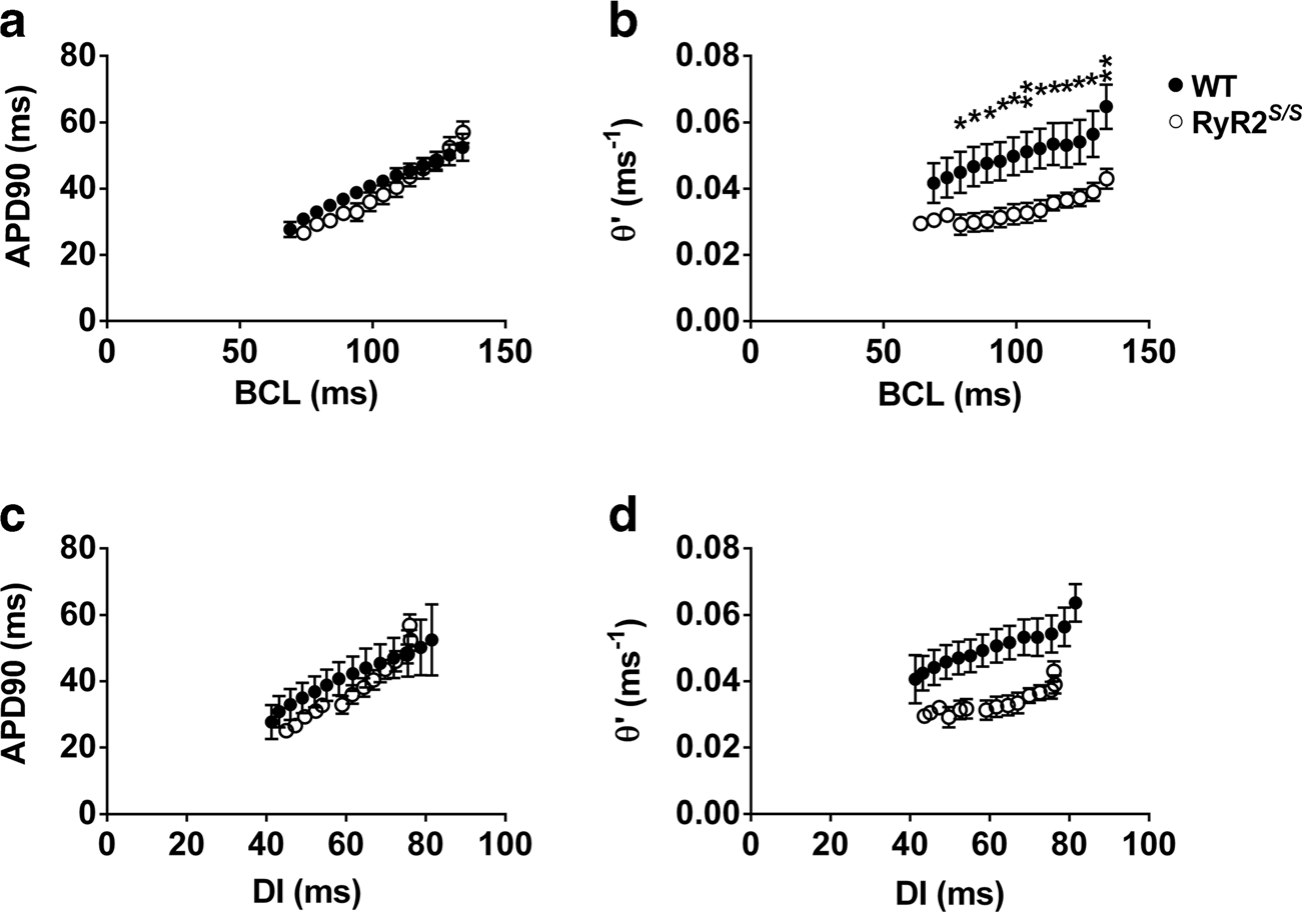
Plots of APD_90_ and *θ*’ at different BCLs and DIs in WT and *RyR2* ^S/S^ hearts. **a, b** Mean (± SEM) values for APD_90_ and *θ*’ respectively at different BCLs (134, 129, 124, 119, 114, 109, 104, 99, 94, 89, 84, 79, 74 and 69 ms) for WT (*n* = 6, *filled symbols*) and *RyR2* ^S/S^ (*n* = 7, *open symbols*) hearts. **c**, **d** Mean (± SEM) values for APD_90_ and *θ*’, respectively, at different DIs for WT (*n* = 6, *filled symbols*) and *RyR2* ^S/S^ (*n* = 7, *open symbols*) hearts. APD_90_ is virtually superimposable at all BCLs between WT and *RyR2* ^S/S^ hearts and thus shows no statistically significant variation. However, *θ*’ is consistently lower in *RyR2* ^S/S^ hearts as compared to WT hearts with significant differences between the genotypes denoted by asterisks; *P < 0.05 and **P < 0.01

Figure 4 plots averaged (mean ± SEM) APD_90_ (A, C) and *θ*’ (=1/latency) (B, D) values in WT (filled symbols) and *RyR2*^S/S^ (open symbols) hearts against BCL (A, B) and DI (C, D). At BCLs, where significant differences between readings at the two genotypes were obtained, this is indicated (*P < 0.05; **P < 0.01). Both WT and *RyR2*^S/S^ showed similar (*P* > 0.05) values of APD_90_ at each BCL and DI. These both declined with decreasing BCL and DI. *RyR2*^S/S^ and WT hearts additionally showed 2:1 block at similar values of BCL (WT 56.5 ± 5.95 ms, *n* = 4; *RyR2*^S/S^: 54 ± 2.5 ms, *n* = 3; *P* = 0.751). As with VERP for the S1S2 protocol, 2:1 block was not obtained from all the mice studied; this was due to arrhythmogenesis warranting termination of the dynamic pacing protocol in four of the seven *RyR2*^S/S^ hearts and two of six WT hearts. In contrast to similar APD_90_, at equivalent BCLs, *RyR2*^S/S^ hearts showed consistently lower *θ* ’ than WT hearts at equivalent BCLs. Indeed, the highest mean *θ*’ showed by the *RyR2*^S/S^ (0.043 ± 0.003 m s^−1^), which was observed at the *highest* BCL, was similar to the lowest *θ*’ (0.042 ± 0.006 m s^−1^) shown by the WT, which was observed at the *shortest* BCLs. These findings were corroborated when the APD_90_ and *θ*’ values were plotted against their preceding DIs reflecting recovery times from the preceding APs (C, D). The present findings demonstrate normal AP repolarization characteristics, but compromised AP conduction in the *RyR2*^S/S^ arrhythmic phenotype, which could arise from abnormalities in gap junction channels and/or Na_v_1.5.

**Fig. 5:**
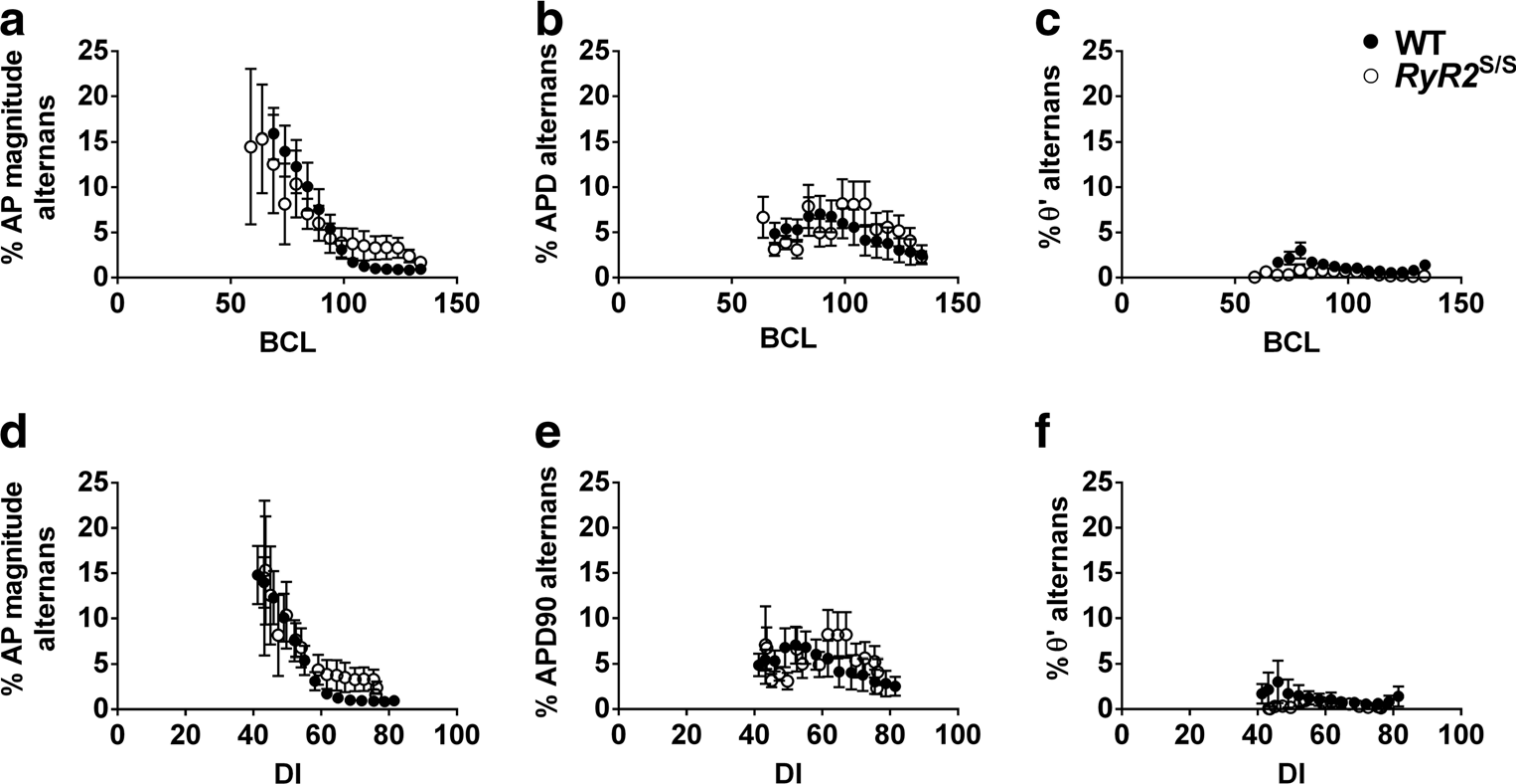
Plots of alternans at different BCLs and DIs. The mean (± SEM) alternans characteristics of AP amplitude (**a**, **d**), APD_90_ (**b**, **e**) and *θ’* (**c**, **f**) for WT (*filled symbols*) and *RyR2* ^S/S^ (*open symbols*) hearts have been plotted as percentage variation between each beat as a function of BCL (**a**–**c**) and DI (**d**, **e**) AP magnitude displays an increasing degree of alternans with decreasing BCL and DI; however, this does not vary between genotypes. Similarly, a small degree of alternans is observed in the APD_90_ and less so the *θ*’ with decreasing BCL and DI, but these do not vary significantly between the WT and *RyR2* ^S/S^ hearts

Alternans of electrophysiological parameters, reflecting temporal variability, often presages arrhythmic activity. Figure 5 assesses the average (mean ± SEM) degree of alternans in AP amplitude (A, D) [30], APD_90_ (B, E) and *θ*’ (C, F) at different BCLs (A-C) and DIs (D-F) in WT (filled symbols) and *RyR2*^S/S^ (open symbols) hearts. Alternans reflects system instability through the mean difference between alternating high and low values of a given parameter normalized to the mean value of the parameter. Both the *RyR2*^S/S^ and the WT demonstrated similarly increasing AP amplitude instabilities with either decreasing BCL or decreasing DI. *RyR2*^S/S^ and WT showed similar APD_90_ and *θ*’ instabilities which similarly varied with decreasing BCL or DI. *θ*’ instabilities were relatively small in contrast to the large changes in their mean values described.

These findings implicate abnormal conduction as opposed to abnormal repolarization in the *RyR2*^S/S^ ventricular arrhythmic phenotype. The underlying mechanism/cause of this abnormal conduction is thus investigated in the next sections.

### Cx43 expression is comparable between the ventricles of WT and *RyR2*^S/S^ murine hearts

Abnormal cardiac conduction can arise from one of three factors: abnormal connexin expression/gap junction formation, impaired Na channel function and/or structural abnormalities such as with fibrosis or hypertrophy. Due to the structural similarity of WT and *RyR2*^S/S^ hearts [49], we pursued the remaining two factors.

**Fig. 6:**
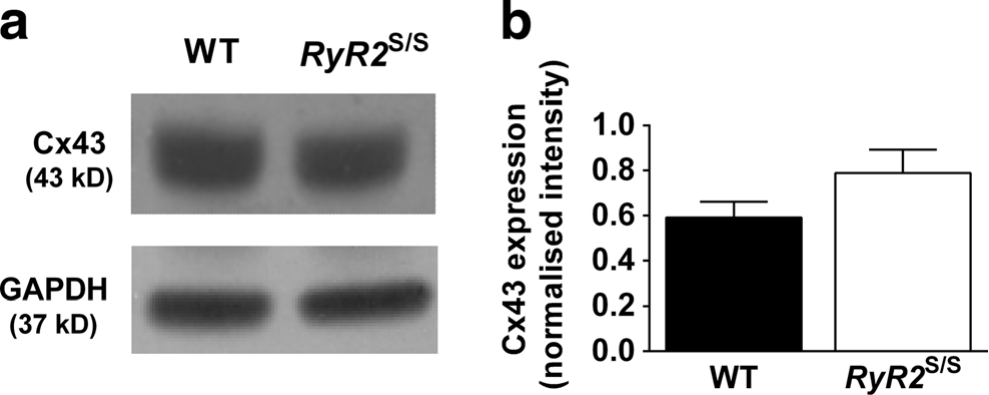
Total Cx43 expression in WT and *RyR2* ^S/S^ ventricles. **a** Representative blots of Cx43 and GAPDH expression in WT and *RyR2* ^S/S^ ventricular tissue and **b** the mean (± SEM) Cx43 expression normalized to GAPDH expression. Cx43 expression was similar between WT and *RyR2* ^S/S^ (0.59 ± 0.07 and 0.79 ± 0.1, respectively; *P* = 0.167, *n* = 4) ventricles

We first assessed the expression of the ventricular gap junction protein, Cx43. Western blots of whole tissue ventricular lysates from WT and *RyR2*^S/S^ hearts demonstrate that the overall expression of Cx43, normalized to the housekeeping gene glyceraldehyde 3-phosphate dehydrogenase (GAPDH), was not significantly different between WT and *RyR2*^S/S^ ventricles (0.59 ± 0.07; *n* = 4 and 0.79 ± 0.1; *n* = 4, respectively; *P* > 0.05, Fig. 6). This suggests that a loss of Cx43 expression is not a contributory factor to the slowed ventricular conduction and increased arrhythmogenesis observed in the ventricles of *RyR2*^S/S^ hearts, in parallel to findings in the atria of the same model [21].

### Decreased Na_v_1.5 expression in the ventricles of *RyR2*^S/S^ murine hearts

**Fig. 7:**
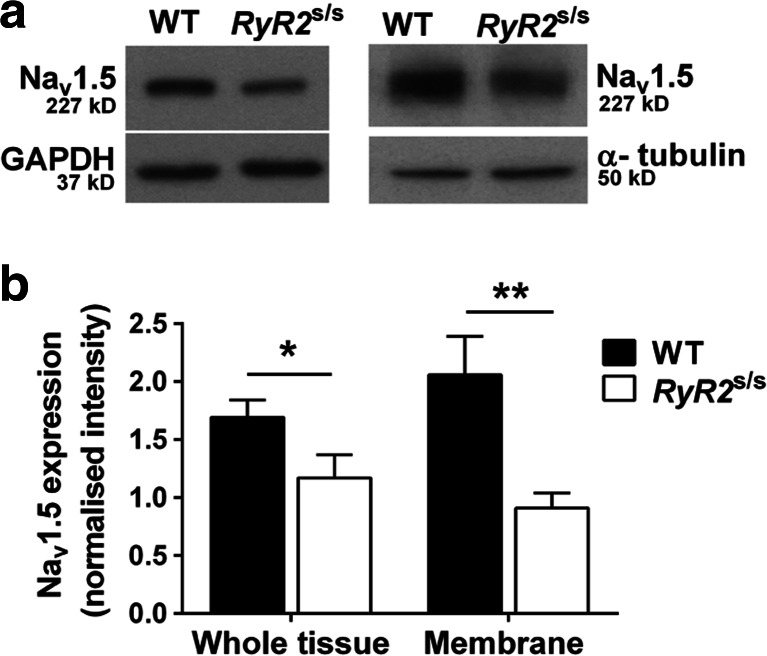
Western blots of Na_v_1.5 expression in whole tissue and membrane fraction samples from WT and *RyR2* ^S/S^ ventricles. Ventricular Na_v_1.5 expression was decreased in *RyR2* ^S/S^ compared to WT, both in the whole tissue (1.17 ± 0.20; *n* = 6, vs 1.69 ± 0.15 *n* = 7, respectively, *P* = 0.048) and in the membrane fraction (0.91 ± 0.13; *n* = 4, vs 2.06 ± 0.33; *n* = 4, respectively, *P* = 0.006). This suggested a greater proportional reduction in membrane relative to total Na_v_1.5 expression in *RyR2* ^S/S^. *Symbols* denote significant differences between genotypes *P < 0.05, **P < 0.01

Western blots of WT and *RyR2*^S/S^ ventricular homogenates (Fig. 7) illustrate a decreased Na_v_1.5 expression in the *RyR2*^S/S^ relative to the WT in both the whole tissue fraction (Fig. 7a; left panel) (1.17 ± 0.2; *n* = 6, and 1.69 ± 0.15; *n* = 7, respectively; *P* < 0.05) and within the membrane fraction (Fig. 7a; right panel) (2.06 ± 0.33; *n* = 4, and 0.91 ± 0.13; *n* = 4, respectively; *P* < 0.01). Thus, Na_v_1.5 expression in *RyR2*^S/S^ ventricles was approximately 69 % of that seen in the WT whole tissue fraction and down to only 44 % of WT in the membrane fraction (Fig. 7b). This significant reduction of Na_v_1.5 expression in the ventricular membrane where the function of Na_v_1.5 channels is crucial to cardiac excitability, and conduction would be expected to lead to a significant reduction in *I*_Na_ in the *RyR2*^S/S^ heart compared to the WT.

### Decreased *I*_Na_ in the ventricles of RyR2^S/S^ murine hearts

**Fig. 8:**
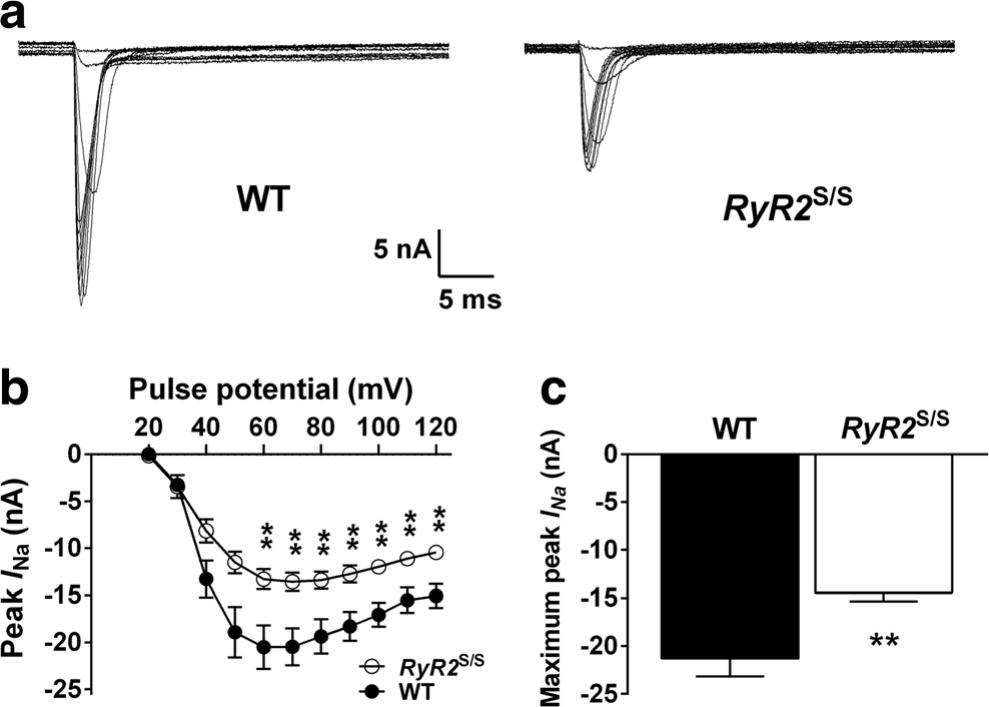
Loose patch clamp recordings of *I*_Na_ activation in WT and *RyR2* ^S/S^ ventricles. **a** Representative currents in response to depolarizing steps increased from 20 to 120 mV in voltage-clamped WT and *RyR2* ^S/S^ ventricular tissue. **b** Peak inward current (mean ± SEM) elicited at each voltage step for WT (*n* = 6) and *RyR2* ^S/S^ (*n* = 12) ventricles. **c** The maximum current recorded during each complete voltage step protocol (mean ± SEM) was larger in the WT than the *RyR2* ^S/S^ ventricles, *P* < 0.0047. The *asterisks* denote significant differences between genotypes of *P* < 0.01

To assess whether the reduced expression of Na_v_1.5 in *RyR2*^S/S^ ventricles correlated with a functional alteration of Na_v_1.5, we measured *I*_Na_ in both WT and *RyR2*^S/S^ ventricles using the loose patch clamp technique. Figure 8a illustrates representative currents elicited by WT and *RyR2*^S/S^ ventricles following a series (20–120 mV voltage excursions) of depolarizing test pulses. The peak current elicited at each voltage excursion and the overall peak current for both WT and *RyR2*^S/S^ ventricles are shown in Fig. 8b, c, respectively. Currents recorded from the WT ventricle were significantly larger than those recorded in the *RyR2*^S/S^ ventricle at depolarizing pulses of 60 mV or greater (*P* < 0.01). The overall peak current in the *RyR2*^S/S^ was −14.45 nA ± 0.88 nA while in the WT it was −21.3 nA ± 1.87 nA (*P* < 0.01); this equates to a 32 % reduction in peak *I*_Na_ in the *RyR2*^S/S^.

## Discussion

The present experiments demonstrate that reduced Na_v_1.5 expression and Na current is associated with the reduced conduction velocity and consequent arrhythmic substrate and ventricular arrhythmogenesis in homozygotic murine *RyR2*-*P2328S* (*RyR2*^S/S^) hearts. The quantitative changes were compatible with earlier reports of linear relationships predicted between the conduction velocity and the peak *I*_Na_ of the AP, but a nonlinear (logarithmic) relationship between peak *I*_Na_ and maximum Na^+^ permeability [20]. Thus, increased arrhythmogenicity was associated with a reduced conduction velocity of ∼22 % during steady 8 Hz pacing and in the region of a ∼33 % reduction during dynamic pacing, which would correspond to comparable reductions in AP wavelength given an absence of significant changes in repolarization characteristics (VERP and APD_90_), and determinants of passive conduction reflected in Cx43 expression. These in turn accompanied reductions in membrane Na_v_1.5 expression of ∼56 % and peak *I*_Na_ of ∼32 %.

The murine *RyR2*^S/S^ heart has proven a useful experimental model for CPVT in reproducing a particular clinically observed human CPVT genotype [25, 40]. *RyR2*^S/S^ ventricular myocytes show features of altered Ca^2+^ homeostasis [15] thought to result from an increased RyR2-mediated Ca^2+^ leak reflecting an increased sensitivity of Ca^2+^ release to cytosolic though not to SR levels [Ca^2+^] [31]. The consequent increase in cytosolic [Ca^2+^] would result in increased sodium-calcium exchanger (NCX) activity whose electrogenic actions would result in triggering events including delayed after-depolarizations leading to ectopic APs that could potentially initiate ventricular arrhythmia. However, these initial studies did not explore for the presence or otherwise for arrhythmic substrate that could sustain the resulting arrhythmia.

Genetic modifications in *RyR2* are also associated with AF phenotypes [17, 31, 37]. This has also been modeled by the *RyR2*^S/S^ system which demonstrates abnormal atrial Ca^2+^ homeostasis, delayed triggering events and atrial arrhythmia [22, 48]. However, they also demonstrated reductions in conduction velocity that could provide an arrhythmic substrate [22]. This was attributed to a reduced Na^+^ current which could be either attributed to a reduced Na_v_1.5 expression or a direct inhibitory effect on Na^+^ channel function of altered Ca^2+^ homeostasis [21]. This could arise from either increased leak of SR Ca^2+^ or the consequently elevated diastolic Ca^2+^. Indeed, recent evidence suggests that altered Ca^2+^ homeostasis can acutely affect cardiac excitability due to both direct [47] and indirect actions on the Na^+^ channel [2, 5, 41]. CaMKII has been shown to directly interact with Na_v_1.5, shifting Na^+^ current availability to a more depolarized membrane potential, thus enhancing the accumulation of Na^+^ channels into an intermediate inactivated state [2]. Increases in CaMKII activity additionally is known to phosphorylate RyR2 which itself increases SR Ca^2+^ leak [45]. Intracellular Ca^2+^ concentration can also acutely modulate Na^+^ current density in ventricular myocytes [5]. Atrial conduction slowing has also been observed in further models of RyR2-mediated Ca^2+^ leak including a CSQ2 mutant [14, 26].

These findings suggest that altered Ca^2+^ homeostasis following the chronic atrial alterations in SR Ca^2+^ release in the *RyR2*^S/S^ system could compromise Na_v_1.5 expression or function as a result of the elevated diastolic Ca^2+^. The present study now demonstrates that *RyR2*^S/S^*ventricles* similarly displayed a reduced Na_v_1.5 expression and consequently reduced peak *I*_Na_, that could explain similar reductions in their conduction velocities [49]. It further extends these findings in localizing this altered expression to the membrane, as well as the whole tissue, fraction (Fig. 7), leading to a reduced maximum rate of AP depolarization, which would be expected to reduce AP conduction velocity, thus creating an arrhythmic substrate. These findings accompanied a greater arrhythmogenicity of *RyR2*^S/S^ murine ventricles, which showed arrhythmic events on extrasystolic (S2) stimulation unlike WT and more frequent arrhythmias that occurred at higher BCLs during dynamic stimulation. These findings took place despite indistinguishable AP recovery characteristics in WT and *RyR2*^S/S^ ventricles, as reflected in VERP and APD_90_ readings, thereby excluding re-entrant mechanisms involving recovery phases of the AP [27]. In contrast, *RyR2*^S/S^ showed reduced indices of conduction velocity, *θ*’ through all BCLs examined compared to WT, despite indistinguishable AP amplitude, APD_90_ and *θ*’ alternans and their variation with BCL or DI, particularly at low BCLs.

Our findings therefore suggest that the arrhythmic substrate results from reduced expression of Na_v_1.5 in the membrane, where a reduced *I*_Na_ leads to slowed AP conduction velocity, in the ventricles of *RyR2*^S/S^ mice. This would be consistent with a situation in which abnormalities in cytosolic Ca^2+^ exert both short- and long-term effects. In the short term, ectopic activity can follow transient elevations in cytosolic [Ca^2+^]. In the long term, chronic elevations in cytosolic [Ca^2+^] can result in a downregulation of either Na_v_1.5 expression or activity, thereby reducing action potential conduction and resulting in arrhythmic substrate. In such a situation, short-term triggering events could potentially form a means for initiating electrical events then perpetuated by the pre-existing arrhythmic substrate. These findings may have broader implications for the mode of therapeutic intervention in a variety of Ca^2+^ dependent, and potentially some Na_v_1.5 dependent, arrhythmia.
